# Transgenic Rat Model of Huntington's Disease: A Histopathological Study and Correlations with Neurodegenerative Process in the Brain of HD Patients

**DOI:** 10.1155/2014/291531

**Published:** 2014-08-03

**Authors:** Yvona Mazurová, Miroslava Anderova, Ivana Němečková, Aleš Bezrouk

**Affiliations:** ^1^Department of Histology and Embryology, Faculty of Medicine in Hradec Králové, Charles University in Prague, Šimkova 870, P.O. Box 38, 500 38 Hradec Králové, Czech Republic; ^2^Department of Cellular Neurophysiology, Institute of Experimental Medicine, The Academy of Sciences, Vídeňská 1083, 142 20 Prague, Czech Republic; ^3^Department of Biological and Medical Sciences, Faculty of Pharmacy in Hradec Králové, Charles University in Prague, Heyrovského 1203, 500 05 Hradec Králové, Czech Republic; ^4^Department of Medical Biophysics, Faculty of Medicine in Hradec Králové, Charles University in Prague, Šimkova 870, P.O. Box 38, 500 38 Hradec Králové, Czech Republic

## Abstract

Rats transgenic for Huntington's disease (tgHD51 CAG rats), surviving up to two years, represent an animal model of HD similar to the late-onset form of human disease. This enables us to follow histopathological changes in course of neurodegenerative process (NDP) within the striatum and compare them with postmortem samples of human HD brains. A basic difference between HD pathology in human and tgHD51 rats is in the rate of NDP progression that originates primarily from slow neuronal degeneration consequently resulting in lesser extent of concomitant reactive gliosis in the brain of tgHD51 rats. Although larger amount of striatal neurons displays only gradual decrease in their size, their number is significantly reduced in the oldest tgHD51 rats. Our quantitative analysis proved that the end of the first year represents the turn in the development of morphological changes related to the progression of NDP in tgHD51 rats. Our data also support the view that all types of CNS glial cells play an important, irreplaceable role in NDP. To the best of our knowledge, our findings are the first to document that tgHD51 CAG rats can be used as a valid animal model for detailed histopathological studies related to HD in human.

## 1. Introduction

Huntington's disease (HD) is an autosomal dominant inherited disorder belonging to the group of systemic brain atrophies. General clinical symptoms are defined by early changes in personality and cognitive functions, followed by typical involuntary choreatic movements; advanced stages of the disease include bradykinesia, rigidity, and dementia. The first symptoms appear frequently between 35 and 50 years of age. The disease is always fatal with an average survival of 10–15 years after the onset of the first symptoms. The disease may develop at any time, even during childhood and adolescence. Of note, juvenile form of HD represents 6% of HD patients [1].

Histopathologically, HD is characterised by premature death particularly of medium-sized (mainly GABA-ergic) striatal neurons but large interneurons are mostly spared (e.g., [2]). Regardless of the development of reactive gliosis within the striatum, loss of the grey matter is extensive and results in the compensatory enlargement of lateral brain ventricles. Due to the general atrophy, the brain weight can decrease by 40% (e.g., [3]).

Severity of the degeneration is evaluated using grading system proposed by Vonsattel and coauthors [2], which classifies HD into five grades (0–4). In grade 0, patients have already clinical symptoms; however, neuronal loss in the head of the caudate nucleus (CN) can be detected only on microscopic level. In grade 1, neuronal loss is accompanied by reactive gliosis, which is evident primarily in the head and tail, less in the body of CN. Grade 2 is characterised by mild to moderated striatal atrophy. In grade 3, due to the progression of atrophy, the medial outline of the CN becomes straight. In grade 4, neuronal loss is a keystone (reaches 95%) and therefore the medial part of the CN is concave. Moreover, brains from patients with late onset of clinical symptoms might show changes occurring in usual aging in addition to those observed in HD (e.g., [2]).

Genetic mutation on short arm of chromosome 4, which causes HD, was discovered in 1983 [4]. Lately, in 1993, gene IT15 (interesting transcript 15), which codes unstable protein huntingtin (htt) comprising variable number of CAG repeats, was identified [5]. Such molecular defect is based on the expansion of this triplet that codes amino acid glutamine. Therefore, HD can be included into the group of polyglutaminopathies.

Mutant form of huntingtin (mhtt) comprises up to 40 repeats and individuals with 36–39 CAG repeats are in risk of developing adult (late-onset) form of HD. Juvenile form of HD develops in patients with 55 and more repeats (e.g., [6]). Although wild-type huntingtin is expressed in all cell types, with the highest concentration in the brain [7], its functions are not yet fully understood [8]. Expanded polyglutamine forms intracellular deposits, particularly in a form of intranuclear and neuropil aggregates (e.g., [8, 9]). It is evident that mhtt displays specific toxicity to striatal (mainly GABA-ergic), less cortical neurons. However, the role of mhtt in the pathogenesis of HD appears highly controversial, ranging from being essential in pathogenesis of the disease (e.g., [6, 8]) to being neuroprotective [10, 11]. Moreover, mhtt also accumulates in nuclei of astrocytes causing their dysfunction, particularly in relation to glutamate uptake. This may further promote vulnerability, especially of striatal medium-sized spiny neurons, to the excitotoxic damage [12]. Therefore, turning our attention to glutamate uptake in astrocytes might be of particular importance for preventing glutamate excitotoxicity in HD [12, 13]. Nevertheless, the presence of mhtt in neural cells is not the only mechanism, which is involved in HD pathogenesis. Metabolic and mitochondrial dysfunctions, oxidative and nitrative stress, and also apoptosis play an important role as well (e.g., [14]).

Generation of transgenic mice as well as transgenic rats advanced significantly the understanding of HD pathology. Indeed, over 20 different rodent models of this disease have been generated to date (for the review see [15]). The firstly introduced transgenic model of HD termed R6 [16] and other generated lines, such as R6/1 and R6/2 (with 115 and 145 CAG repeats, resp.), belong to the most frequently used models of HD (e.g., [17, 18]). These models of HD are characterized by early onset and fast progression of behavioural deficits (later also by motor dysfunction) and presence of intranuclear polyglutamine (polyQ) inclusions (4 weeks postnatally), but without the evident reduction of neurons even in aged mice [16, 19]. Therefore, they simulate rather juvenile than adult form of HD.

In 2003, von Hörsten and coworkers generated the first transgenic rat model of HD (tgHD51 rats; Sprague-Dawley background), which carries a truncated htt cDNA fragment with 51 CAG repeats under the control of the native rat htt promoter [20]. Due to relatively smaller number of CAG repeats, this model exhibits a high degree of similarity to the late-onset form of HD. These rats survived up to 24 months and exhibited slow cognitive decline and not as much of motor deficit (e.g., [9, 20–24]). Neuropil aggregates of polyglutamine and typical intranuclear inclusions in neurons appeared in the brain approximately at 6–9 months of age [9]. Despite the fact that tgHD51 rats displayed enlarged lateral brain ventricles, stereological analysis revealed only a subtle decrease in the number of neurons in the striatum, while no changes were observed in number of neurons in the frontal cortical layer V of 12-month-old tgHD51 rats when compared with age-matched wild-type controls [24].

Astrocytes are the most numerous type of glial cells in mammalian CNS. Currently, they are considered as highly active cellular component of the CNS parenchyma with functional pleiotropy essential for neuronal survival and function (e.g., [25–27]). Besides others, they are closely associated with neuronal synapses (“tripartite synapse”—[28]), secrete and degradate proteins of extracellular matrix, and are responsible for uptake and release of different neurotransmitters, primarily of the glutamate. It has been suggested that enhanced release of glutamate and other substances may represent an early event in a number of, if not all, neurodegenerative diseases (e.g., [29]).

The communication between neurons and local blood flow mediated by astrocytes is elementary for the maintenance of functional microenvironment in the grey matter of the entire CNS parenchyma; therefore the term neuronal-glial-vascular unit is used [30]. On the other hand, under pathological conditions, the perivascular end-feet can restrict transport or diffusion across the blood-brain interface [31].

Glial fibrillary acidic protein (GFAP [32]) is essential for immunohistochemical identification of astrocytes. However, GFAP is densely expressed only within the cell body and larger processes of astrocytes, unlike the numerous fine processes representing the majority of the total volume of the astrocytes, which are GFAP-negative [30]. Any type of the CNS injury (primarily the acute damage) initiates morphological changes of some astrocytes, which become reactive (e.g., [33]). They are hypertrophic with longer and thicker main processes and increased expression of GFAP due to the formation of bundles of gliofilaments (e.g., [34]). In the acute phase of reaction, astrocytes also reexpress intermediate filaments significant for glial precursors—nestin and vimentin (e.g., [35, 36]).

Beta-subunit of Ca^2+^ binding protein (S100*β*) is another putative astrocytic marker (e.g., [37]). S100*β* is produced, stored, and released primarily by astrocytes; however, it is also expressed by many other cells in the CNS and other body regions. S100*β* is localized in the cytoplasm and nucleus and involved in the regulation of a number of cellular processes (e.g., [38, 39]). Regardless of large body of studies, only few of them are dealing with morphology of S100*β* ^+^ cells (e.g., [39, 40]). Most of experimental or clinical studies are related to detection of concentrations of S100*β* within the tissue, or its plasma (or CSF) levels, whose changes are significant for various diseases, probably due to release of S100*β* from damaged astrocytes (e.g., [38]).

NG2 glia (polydendrocytes or synantocytes) represent a fourth type of glia in the CNS (e.g., [41]). They exist abundantly in both grey and white matter of the mature CNS in rodents as well as human. They constitute the major group of cells undergoing mitosis in the adult rodent brain and are almost as numerous as astrocytes [42]. NG2 cells are primarily described as the precursors of myelinating oligodendrocytes (OLPs). However, many of the NG2 cells remain in the NG2-positive state for a significant time and have a unique capacity to communicate with nearby cells, forming multiple contacts with astrocytes, microglia, oligodendrocytes, and even neurons [43]. In human brain, significant morphological changes related to the progression of pathology were studied particularly in multiple sclerosis and gliomas [44].

Microglia, the immunocompetent highly motile cells of the CNS, are extremely plastic and undergo a variety of structural changes based on their location and current role [45]. In the grey matter, the most frequent is ramified form, which express protein Iba1 (ionized calcium-binding adapter molecule 1) also known as AIF-1 (allograft inflammatory factor 1). The density of this marker significantly increases with activation of cells. It is obvious that the activation of microglia is a basic mechanism in the defence of the CNS, also in relation to neurodegenerative processes (e.g., [45, 46]). Although the role of microglia in neurodegeneration is still controversial, it is evident that in human brain they are activated in early stages of NDP of different phenotype, primarily in HD (e.g., [46, 47]), Parkinson's disease (e.g., [48]), and Alzheimer's disease (e.g., [49, 50]). It is possible that microglia transform to phagocytes and target neurons as the disease progresses but appear to be dysfunctional with increasing amounts of ingested debris [48].

It is commonly known that the neurodegenerative process of HD phenotype is a chronic process, morphologically characterized by the progressive degeneration of neurons, principally in the striatum, but gradually affecting almost all parts of the brain. This results in a reduction of grey matter and brain atrophy with compensatory enlargement of the lateral brain ventricles. Nevertheless, also as the second component of the brain parenchyma, the glial cells play an irreplaceable role in this process. The reaction of astrocytes to any damage of the CNS parenchyma in a sense of their conversion into the reactive intensely GFAP-positive subset is well known already for long time. Although the participation of other types of glial cells, particularly of microglia and NG2 glia, in neurodegenerative process has been studied in last two decades, the histopathological interrelations among all above-mentioned cell types have not been well described yet. Moreover, the validation of existing transgenic rat model of HD51 from this point of view is still lacking.

## 2. Materials and Methods

All animal procedures were performed in accordance with the directive of the EEC (86/609/EEC) and the use of animals in our experiments was reviewed and approved by the Animal Ethical Committee of Charles University in Prague, Faculty of Medicine in Hradec Králové.

### 2.1. Animals

Male homozygous tgHD51 CAG rats (+/+; *n* = 18) and their wild-type (wt) littermates (−/−; *n* = 18) were obtained from H. P. Nguyen and O. Riess, the Department of Medical Genetics, University of Tübingen, Germany. Rats were sacrificed at the age of 2 or 3 months (tgHD *n* = 3; wt *n* = 2), 6 months (tgHD *n* = 2; wt *n* = 3), 12 months (tgHD *n* = 4; wt *n* = 5), 18 months (tgHD *n* = 5; wt *n* = 4), and 22–24 months (tgHD *n* = 4; wt *n* = 4).

### 2.2. Postmortem Specimens

The brains of three patients with approximately 2-, 8-, and 20-year clinical manifestation of HD (sex/age: ♀/52, ♂/38, and ♀/52) were studied. Three control brains of patients (sex/age/weight: ♂/33/1440 g, ♀/43/1510 g, and ♂/56/1400 g) with no history of neurologic disorder or brain lesion were also taken for the study. The clinical features of HD (described in autopsy records) were characteristic for the given stages in all three patients. However, detailed neurological records or results of genetic testing were not available because old archival material was used. Surprisingly, the total brain weight was markedly reduced in all HD cases independent of the sex and duration of the illness (duration/sex/weight: 2 y/♀/1160 g, 8 y/♂/1150 g, and 20 y/♀/1120 g) in comparison with control human brains. The severity of striatal histopathological changes was graded (grades 0–4) according to Vonsattel and coauthors [2].

Paraffin blocks of brain tissue from autopsies were taken from the neostriatum (the caudate nucleus and putamen) at the level of the globus pallidus and at the level of the nucleus accumbens. The blocks were donated by Fingerland's Department of Pathology, Faculty Hospital in Hradec Králové.

### 2.3. Histology and Immunohistochemistry

Animal brains were processed either with formalin fixation (4% neutral formaldehyde) and embedded in paraffin or with 4% paraformaldehyde (in 0.1 M phosphate buffer) fixation and frozen sections preparation. The transcardial perfusion with fixative solution under deep anaesthesia followed by postfixation (for 3 days or 2 hours, resp.) was made in all animals. Brains were transversely cut using the Brain Blocker (Better Hospital Equipment Corp., USA) to obtain the identical blocks of brain tissue. After postfixation, the brain hemispheres were separated and processed separately.

#### 2.3.1. Paraffin Sections

Histological processing was the same for both the experimental material and autopsies. Serial coronal sections (7 *μ*m thick; 120 sections per each rat brain hemisphere), prepared by conventional histological processing, allowed us to study the same region with different antibodies. Findings obtained by immunofluorescent detections (double-labelling) were mostly confirmed by a single antibody detection using peroxidase-antiperoxidase (PAP) immunohistochemistry on parallel paraffin sections.

#### 2.3.2. PAP Immunohistochemical Detection

For immunohistochemical detection, deparaffinized and rehydrated sections were used. Pretreatment in microwave 3 × 5 min at 800 W in the sodium citrate buffer (pH 6.0) and washing in 0.01 M PBS was mostly performed. For detection of polyglutamine deposits the pretreatment with 98% formic acid (5 min at room temperature) was required. Incubation in blocking solution (water solution of H_2_O_2_) for 20 min was followed by incubation with primary antibodies (Table 1) performed overnight at 4°C. Sections were then washed and incubated with the appropriate biotinylated secondary antibody (Jackson ImmunoResearch Lab., USA, 1 : 500) for 45 min at room temperature and subsequently with a streptavidin conjugate of peroxidase (Dako, CR) also for 45 min. Visualization of bound antibody was performed using DAB (Sigma-Aldrich, CR) and H_2_O_2_.

#### 2.3.3. Cryostat Sections

Postfixed blocks of rat brains were placed stepwise in solutions with gradually increasing sucrose concentrations (10%, 20%, and 30%) for cryoprotection at 4°C. Serial coronal cryostat free-floating sections (30 *μ*m thick) were cut. The slices were incubated with 5% Chemiblocker (Millipore, MA, USA) and 0.2% Triton in PBS at 4°C.

#### 2.3.4. Immunofluorescent Double-Labelling Technique

The sequential technique for immunofluorescent double-labelling of antibodies (Ab) was same for both types of sections. Avidin or appropriate secondary Ab was labelled with Cy-3 or Alexa Fluor 660 (red) and with Alexa Fluor 488 or 594 (green) and nuclei were counterstained with DAPI (blue). The negative control, omitting the primary antibody, was made in each labelling.

Photomicrographs were made with Lucia G/F software version 4.82 (Laboratory Imaging, Prague, Czech Republic) or Quick Photo Camera 2.3 software (Promicra, Prague, Czech Republic).

### 2.4. Quantitative Analysis

In order to characterize the progression of NDP in the striatum of tgHD51 rats, we used the quantitative analysis of the median diameter of neuronal nuclei as a marker of proposed significant process in a course of neurodegeneration in tgHD rats; it means the shrinkage of striatal neurons. We would like also to determine the onset of significant neuronal degeneration in the striatum of tgHD51 rats and the possible participation of age-related changes.

The region of rat brain, used for analysis, was determined according to the brain atlas [51] from the level 14 (+0.95 from bregma) dorsally in a sequence of 3 slices, each 7 *μ*m thick, in the distance of 15 sections (i.e., approximately 105 *μ*m); it means that analysed part of rat brain was of about 220 *μ*m thick. Selected sections were labelled with NeuN antibody, which marks selectively the nuclei of mature neurons, using the PAP immunohistochemical detection.

The groups of tgHD and wt rats were divided into two basic subgroups of “young” 3- and 6-month-old (0–6 months) and “old” 12-, 18-, and 24-month-old (>6 months) rats. The number of analysed sections was the following: 6 sections in the “young_wt” group, 6 sections in the “young_tgHD” group, 18 sections in the “old_wt” group, and 24 sections in the “old_tgHD” group. Neuronal nucleus median diameter was obtained from 50 independent measurements in the central area of the striatum on each analysed section. Due to possible distortions of the shape of neuronal nucleus in the section, the largest size of the nucleus was considered the nucleus diameter. Each group of rats of the same age was represented by the set of all medians in given group.

### 2.5. Statistical Analysis

Statistical analyses of the differences between groups were performed using MS Excel 2007 (Microsoft Corp., Redmond, WA, USA) and NCSS 2007 (NCSS LLC, Kaysville, Utah, USA). The median diameters were compared using Kruskal-Wallis Multiple-Comparison *Z*-Value Test (Dunn's Test). The test with Bonferroni correction was used to adjust multiple comparisons with the family-wise *α* at 0.05.

## 3. Results

In tgHD51 rats, the NDP in the striatum starts to develop only after 6 months of age. Surprisingly, the most distinct changes in striatal grey matter develop by the end of the first year of age (probably between 9 and 12 months). The end of the first year represents the turn in the development of morphological changes related to the progression of NDP within the striatum of tgHD51 rats. These findings correspond to the course of HD in human brain, where the motor and behavioural changes precede the loss of striatal neurons [20]. We demonstrate possible parallels between the HD progression in humans and the above-described transgenic rat model and prove the validity of our findings for human HD pathology. The cases demonstrated here represent the sequence of 3 stages (grades 1-2, grade 3, and grade 4) of the progression of HD in human brain.

It is almost impossible to dissociate the alterations referring to neurons and glia in a course of NDP because of very close relationship and mutual influence of both main components of striatal parenchyma. However, we would like to stress some features specific for each of them in a course of the development of NDP within the striatum of both rat and human brains. For that reason, we described their involvement in progression of NDP separately.

### 3.1. Neuronal Degeneration during the Progression of HD

**Figure 1:**
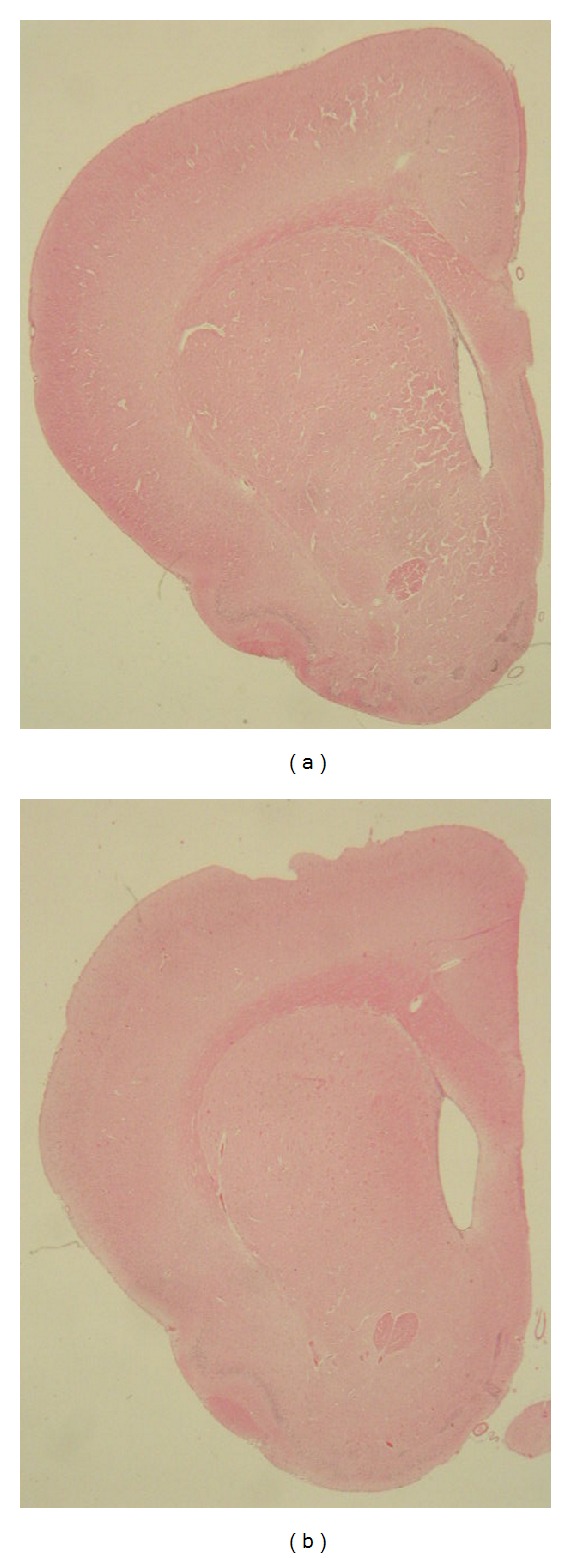
(a) Lateral brain ventricles are quite narrow in young (3-month-old) control wt rats, unlike (b) notably enlarged ventricles in 18-month-old tgHD51 rats, owing to the progression of striatal atrophy which confirms the development of NDP.* Haematoxylin and eosin*.* Direct magnification *1.5x.

When we compare the brains of 2- and 3-month-old, (young adult) wild-type and tgHD rats, there is no difference in morphology of the striatum. Also lateral brain ventricles are narrow, of the same shape in both mentioned groups (Figure 1(a)). Only in 12-month-old tgHD51 rats appears the identifiable enlargement of lateral ventricles, which documents developing striatal atrophy. The process gradually progresses resulting in prominent widening of lateral ventricles (Figure 1(b)), with concave medial outline of the striatum in the oldest (22–24-month-old) tgHD rats, which is fully comparable with the progression of HD in human brain.

#### 3.1.1. Degeneration of Striatal Neurons

**Figure 2:**
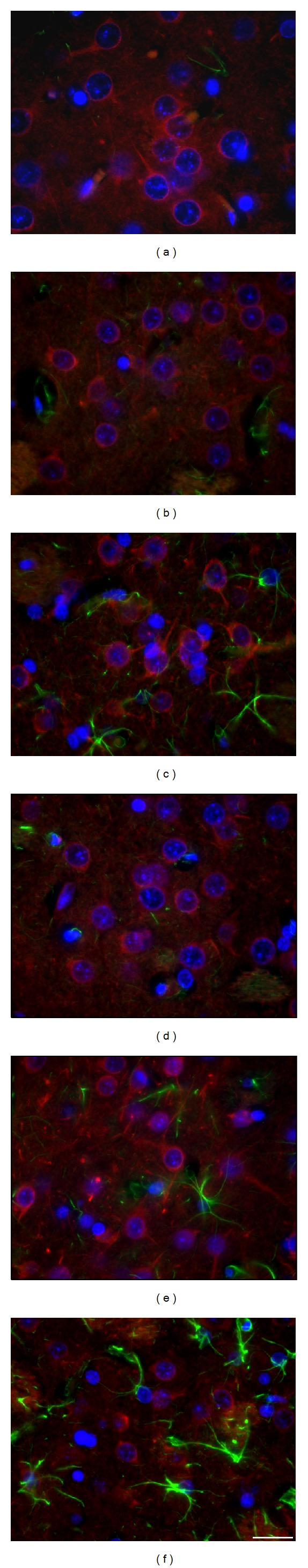
Significant decrease in the size (diameter) of the striatal neurons in a course of the development of NDP is marked by accompanied changes in the diameter of their nuclei, due to the maintenance of the nucleo-cytoplasmic rate. Detection of the *β*-III-tubulin^+^ neurofilaments, which fill in the entire cytoplasm of neuronal body and processes, enables to document the shrinkage not only of neuronal nuclei but also of the bodies of neurons, due to either physiological ageing process (a–c) or that caused by the progression of neuronal degeneration in tgHD rats (d–f). The number of GFAP^+^ astrocyte grows up only slightly in course of ageing (a–c), but significant increase (astrogliosis) is evident in oldest tgHD rats (f). (a) wt—2-month-old, (b) wt—12-month-old, (c) wt—22-month-old, (d) tgHD—6-month-old, (e) tgHD—12-month-old, (f) tgHD—24-month-old rats.* Anti-*β*-III-tubulin (red) + anti-GFAP (green) + DAPI (blue)*.* Bar *20 *μ*m.

**Figure 3:**
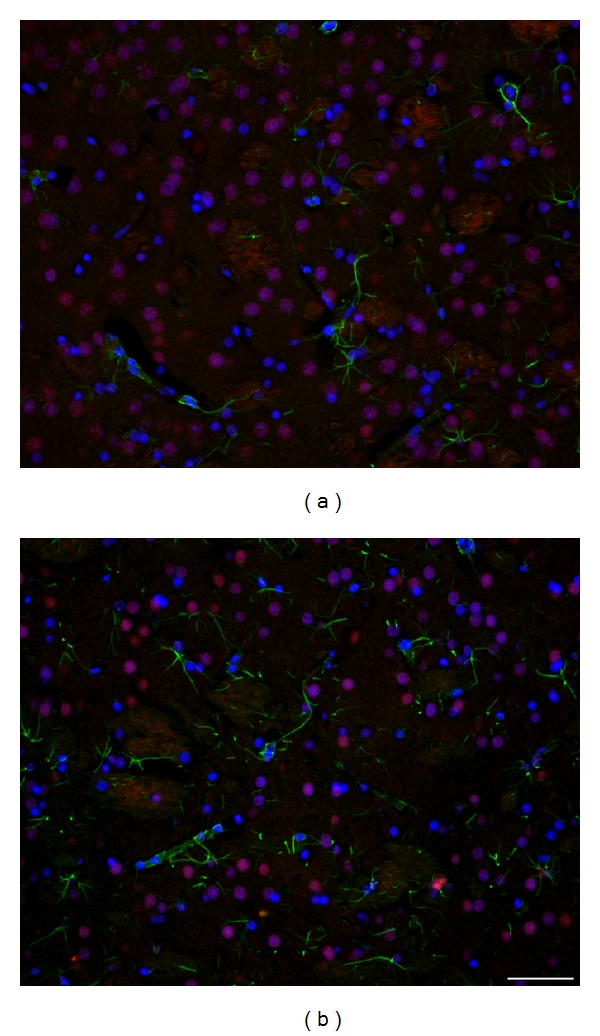
Significant difference in the number and size of neurons/neuronal nuclei (NeuN^+^) is evident if we compare (a) 2-month-old wt rats and (b) 18-month-old tgHD rats; (b) concomitant reactive astrogliosis is already developed in 18-month-old tgHD rats; it is also apparent that the degeneration of neurons in tgHD rats is typically selective (alike in human HD brain).* Anti-NeuN (red) + anti-GFAP (green) + DAPI (blue)*.* Bar *50 *μ*m.

**Figure 4:**
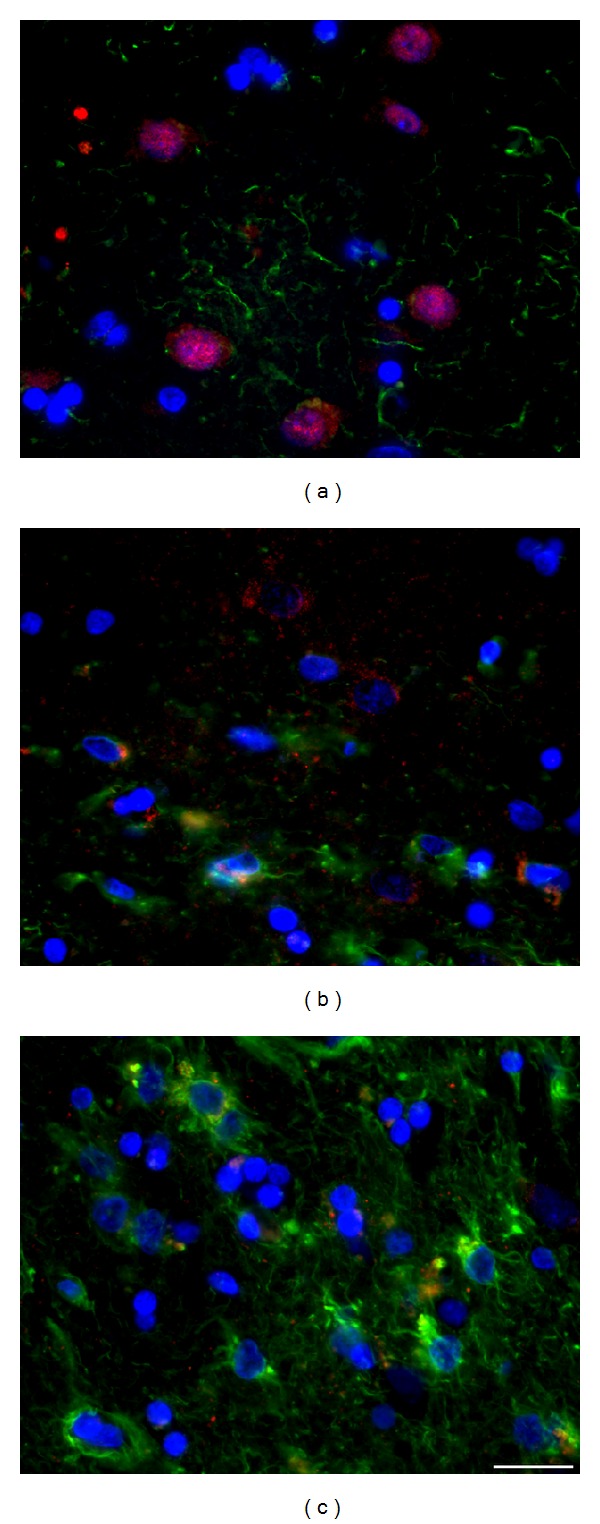
In postmortem samples of human HD brain, (b, c) gradual progression of chronic striatal NDP marked by massive neuronal degeneration and severe concomitant astrogliosis is evident in comparison with (a) intact control brain. (a) Control (♂/56), (b) HD duration 8 years, grade 3 (♂/38), and (c) HD duration 20 years, grade 4 (♀/52).* Anti-NeuN (red) + anti-GFAP (green) + DAPI (blue)*.* Bar *20 *μ*m.

Striatal neurons (*β*-III-tubulin^+^/MAP2^+^) or their nuclei (NeuN^+^ or DAPI^+^) form typical clusters in all tested groups of wt (e.g., Figures 2(a), 2(b), and 3(a)) and tgHD rats (e.g., Figures 2(c)–2(f) and 3(b)) and in human postmortem samples of intact striatum (Figure 4(a)). However, with the progression of HD in humans, gradual decrease in number of neurons is significant, particularly in advanced stages of HD (grade 3—Figure 4(b) and grade 4—Figure 4(c)).

Nuclei of striatal neurons are very characteristic, especially due to their large size and fine loosely arranged chromatin in comparison with significantly smaller nuclei with more densely arranged chromatin of glial cell. Despite the fact that striatal neurons become gradually smaller in course of HD progression (compare Figures 3(a) and 3(b)), such specific features of neuronal and glia nuclei always enable their distinguishing. Our immunohistochemical analysis of neuronal nuclei by NeuN shows slow but already significant progression of neuronal degeneration in the striatum from 12 to 24 months of age of tgHD rats (Figure 3(b)) when compared with age-matched controls (Figure 3(a)) and younger tgHD rats. The most typical for NDP in tgHD51 rats is a gradual decrease in size of neuronal bodies and nuclei (with maintenance of nucleo-cytoplasmic rate), which results in the disintegration and disappearance of affected neurons (Figures 2(d)–2(f)), ultimately scavenged by microglia (Figures 12(b) and 12(c)).

In the human HD brain, grades 1-2 (with approximately 2-year clinical manifestation), the degeneration and loss of neurons were only random; therefore, the loosening of the neuropil has not been apparent yet. On the other hand, in grade 3 (approximately 8-year clinical history), neuronal degeneration was already obvious (Figure 4(b)). Depletion of neurons (particularly in the CN and putamen (Pu)) accompanied with rarefaction of the neuropil resulted in a reduction of striatal volume and noticeable enlargement of the lateral ventricles. In grade 4 (with approximately 20-year clinical diagnosis) the entire corpus striatum (CN, Pu, and globus pallidus) was affected by degeneration of neurons and neuropil (resulting in severe striatal atrophy) and therefore the concomitant astrogliosis here prevailed (Figures 4(c), 10(b), and 10(c)). The remaining striatal neurons (marked by their prominent nuclei) gradually became smaller with the progression of NDP, like in the brain of tgHD rats.

Additionally, we confirmed that, alike in human HD brain, neuronal degeneration is selective, that is, affecting primarily certain groups of neurons in the striatum of investigated senescent tgHD rats and moreover that age-related changes contribute to final extent of NDP.

#### 3.1.2. Quantitative Analysis of Neuronal Degeneration in the Striatum of tgHD Rats

We supposed that neuronal degeneration in the striatum of tgHD rats manifests primarily by the decrease in a volume/size of neuronal bodies including their nuclei. In order to precisely characterize the progression of NDP within the striatum of tgHD51 rats, our morphological findings were supplemented by quantitative analysis of the diameter of neuronal nuclei labelled with NeuN. Also the proportion of age-related changes in this process was assessed.

**Figure 5:**
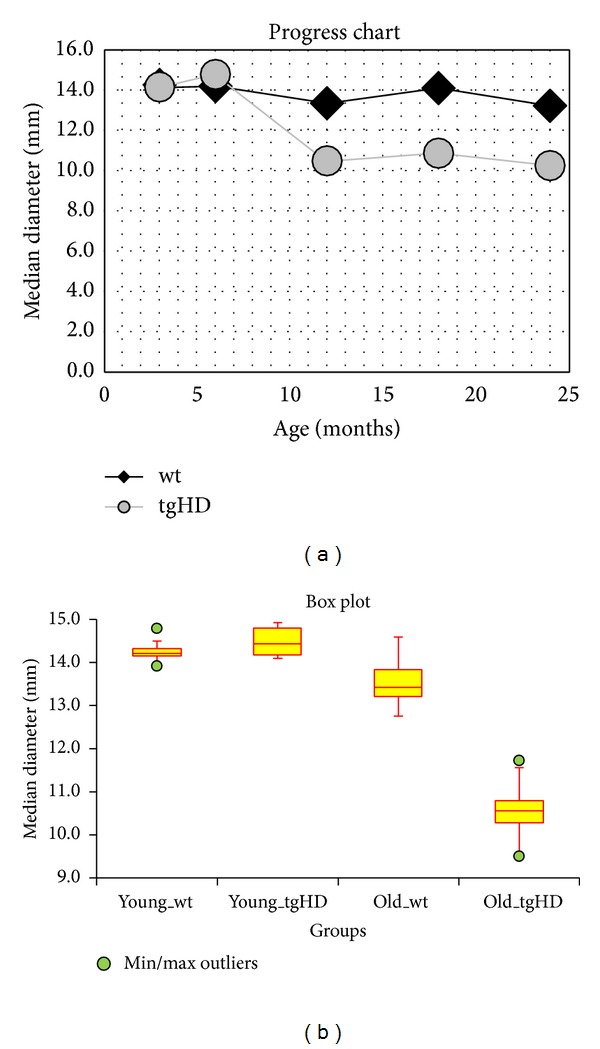
(a) Progress Chart: the progression in decrease of the median diameter of neuronal nuclei with age of rats in wt groups (black line) and tgHD51 rats (grey line). (b) Box Plot: statistical characteristic of the groups of rats. The multiple comparison of the median diameter of neuronal nuclei of the groups of rats: “young_wt” and “young_tgHD” are groups of rats 3 and 6 months (0–6 months) old; “old_wt” and “old_tgHD” are groups of rats 12, 18, and 24 months old (>6 months).

The progression in decrease of the median diameter of neuronal nuclei with age of rats in both wt and tgHD groups of rats is documented by Progress Chart (Figure 5(a)). In the first two groups of rats, that is, 2-3-, and 6-month survivors, no differences in the median diameter of NeuN-positive nuclei were detected when compared within the individual group or among the groups. Striking decrease in the median diameter of neuronal nuclei by 26% was detected in 12-month-old tgHD rats unlike the parallel wt group, in which the decrease was only 7.6%, when compared with the youngest animals in both mentioned groups. Surprisingly, further progression in decrease of the median diameter of neuronal nuclei was not so rapid; however, finally the reduction reached 28.7% in 24-month-old tgHD rats, when compared with age-related degeneration in 24-month-old wt rats (only 8.3%).

Statistical characteristic of the groups of rats using Box Plot (Figure 5(b)) enables the multiple comparison of the median diameter of neuronal nuclei of the following groups of rats: “young _wt” and “young_tgHD” are groups of rats 3 and 6 months (0–6 months) old; “old_wt” and “old_tgHD” are groups of rats 12, 18, and 24 months old (>6 months). Results of Kruskal-Wallis Multiple-Comparison *Z*-Value Test (Dunn's Test) of the mentioned groups of rats (Table 2) indicate significantly different pairs of groups (Bonferroni test: medians are significantly different if *Z*-value is > 2.6383). It is evident that the median diameter of neuronal nuclei in “old_tgHD” group is significantly statistically different from all other evaluated groups. Differences among three remaining groups are statistically insignificant.

We can conclude that the rate of neuronal degeneration reaches maximum at the end of the first year of animal's age and then, in the following 12 months, it proceeds rather slowly. Moreover, it is potentiated with age-related changes particularly in the oldest animals. Unexpectedly, the transitional amelioration of the process up to slight improvement appeared in both groups (wt and tgHD) of 18-month survivors. Neuronal degeneration in wt rats can be attributed only to the debit of the aging process; the decrease in size of nuclei was slow and the difference between 2-3-month-old rats and 24-month-old ones was only 8.3% (Figure 5(a)).

#### 3.1.3. Rarefaction of Neuropil and Alterations in Morphology of Synapses

**Figure 6:**
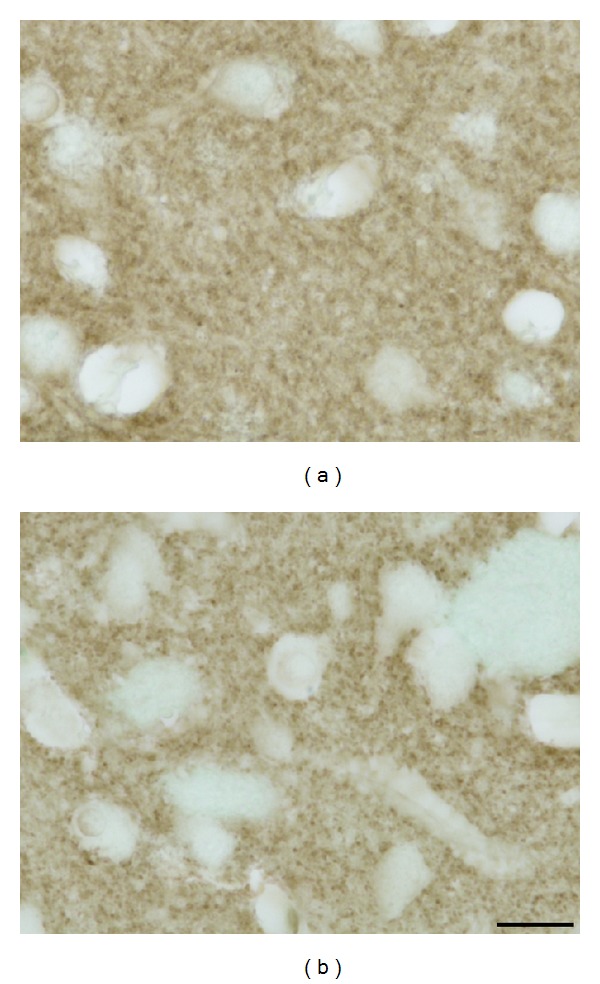
(a) In young control animals (2 months old), synapses within the neuropil are very numerous, fine and of uniform size; (b) the most conspicuous alteration in old (here 18 months) tgHD rats is variable size and enlargement (coarsening) of most of synapses; however, also their decreased number participating in loosening of neuropil is evident.* Anti-synaptophysin counterstained with *0.1%* methyl green*.* Bar *10 *μ*m.

**Figure 7:**
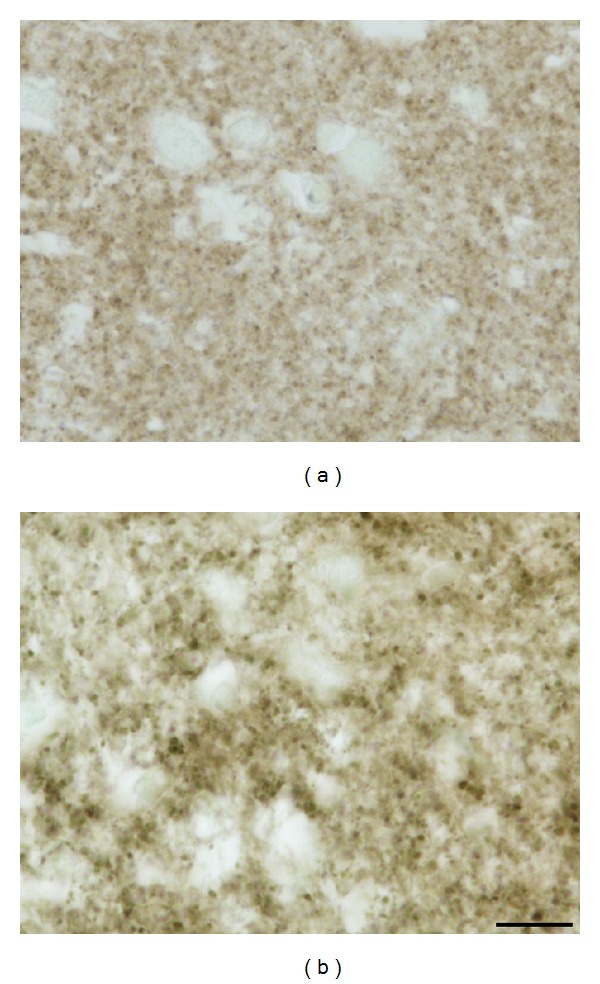
(a) Synapses in intact (control, ♂/56) human brain are (like in rats) uniform and densely accumulated within the neuropil; (b) they also become coarser and of variable size with the progression of NDP in HD patients (here ♀/52, grade 4, duration 20 years); continuous decrease in their number significantly participates in rarefaction of the neuropil, most prominent in terminal stage of NDP.* Anti-synaptophysin counterstained with *0.1%* methyl green*.* Bar *10 *μ*m.

**Figure 8:**
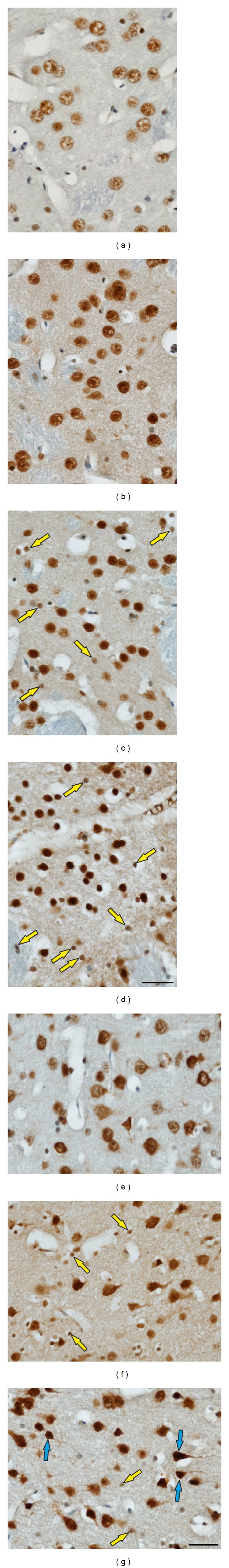
(a) Only fine polyQ deposits are spread in the nuclei of striatal neurons in control 6-month-old rats unlike (b) a higher density of polyQ deposits in age-matched tgHD rats; (c) significantly increased density of polyQ inclusions, in both neurons and some glial cells* (yellow arrows)*, is already in 12-month-old tgHD rats and (d) particularly in aged (here 18-month-old) tgHD rats; increased density of polyQ expression is probably also influenced by the shrinkage of nuclei during the degeneration of neurons; the progression of rarefaction of the neuropil is also apparent. (e) In the cortex of 2-month-old wt rats, perinuclear polyQ positivity is only in some neurons, unlike increased concentration of polyQ (mhtt) deposits particularly within the cytoplasm, primarily of pyramidal neurons, in (f) 12-month- and (g) 18-month-old tgHD rats; in aged tgHD rats, the significant number of degenerated (hyperchromic) neurons is present* (blue arrows)*; only few polyQ^+^ and subsequently degenerated glial cells* (yellow arrows)* and particularly loosening of the neuropil are also characteristic for the progression of NDP in the cortex of tgHD51 rats.* Anti-polyQ counterstained with *0.1%* methyl green*.* Bar *20 *μ*m.

Striatal atrophy, in the case of HD, is primarily caused by the degeneration of striatal neurons. Of course, the most prominent feature, seen on histological preparations, is a gradual reduction of neuronal bodies marked by the nuclei. Indeed, the reduction in a volume of neuropil is at least of the same importance. Large amounts of dendrites (with dendritic spines) extending from neuronal bodies (labelled with anti-MAP2—Figure 12, less with anti-*β*-III-tubulin—Figure 2) with myriads of synapses represent the essential component of the neuropil. Although the rarefaction of neuropil is not based only on the degeneration of this network of neuronal processes and synapses, it demonstrates the progression of such process in both human and rat brains (Figures 8(a)–8(d)). In addition, we also proved the alterations in a character of synapses. In control brains of both rats and humans, synaptophysin-positive synapses are very fine, of uniform size and shape, and plentiful (Figures 6(a) and 7(a)). With the progression of NDP, most of synapses become coarser, more prominent, but of variable size, and some of them are intensely labelled for synaptophysin; consequently, their number gradually decreases (Figures 6(b) and 7(b)). Despite different size of synapses in rat (Figures 6(a) and 6(b)) and human (Figures 7(a) and 7(b)), the mentioned alterations are of the same character. Since the severity of the striatal damage is also influenced by duration of NDP, the changes in morphology of synapses, and particularly the loosening of neuropil, are certainly more prominent in advanced stages of HD in human brain (Figure 7(b)) than in terminal stage of NDP in tgHD rats (Figures 6(b) and 8(d)). Additionally, the alterations in glial component and the ageing-associated changes (see Section 3.2.) also markedly influence loosening of the neuropil. Indeed, the pattern of such process in this basic aspect is the same for both tgHD rats and HD patients.

#### 3.1.4. Polyglutamine Deposits

Detection of polyglutamine deposits using polyQ-huntingtin provides interesting findings, which give a complete histopathological picture of HD progression. In wt rats, polyQ detects a normal polyglutamine domain huntingtin encoded by lower number (about 35 or less) of consecutive glutamine repeats; therefore, only fine polyQ deposits are spread in the nuclei of striatal neurons (Figure 8(a)). In contrast, the pathogenic alleles usually contain 39 or more glutamine repeats, which results in production of mhtt and increased density of intranuclear polyQ expression in relation to the progression of NDP of HD phenotype (Figures 8(b)–8(d)). It is necessary to emphasize that increased density/concentration of intranuclear polyQ deposits is also potentiated by the shrinkage (decreased volume) of nuclei in degenerated neurons in course of the progression of NDP (compare Figures 8(a) and 8(b)–8(d)). Similarly, the number of affected polyQ^+^ glial cells significantly increases with advancing NDP (Figures 8(c) and 8(d)), most likely in relation to the development of reactive astrogliosis; that is, mhtt is probably preferentially expressed by reactive astrocytes. Surprisingly, using polyQ-huntingtin antibody, neither typical large intranuclear nor neuropil aggregates were seen. Moreover, neurons in adjacent cortex also exhibit intranuclear but more cytoplasmic polyQ deposits; therefore, they are more densely stained in comparison with striatal neurons, particularly in tgHD rats (Figures 8(e)–8(g)). On the contrary, only few cortical glial cells express polyQ, which corresponds to the absence of typical reactive gliosis in this region. The presence of very densely labelled (hyperchromic) degenerated/shrunk cortical (primarily pyramidal) neurons—demonstrated also by other histological staining methods—unequivocally confirms that, in tgHD51 rats, NDP in the striatum is accompanied with the degeneration of cortical neurons (Figures 8(f) and 8(g)) alike in human HD brain.

Moreover, the highest density of deposits in shrunk/hyperchromic terminally degenerated neurons (less prominent in glial cells) corresponds to the hypothesis that accumulation of mhtt results in the cell death.

### 3.2. Glial Cells during Progression of HD

It is evident that the developments of changes in glial cell morphology, and certainly also in their function, are conditioned by the intensity and rate of neuronal degeneration in the context of the neuron-glia relationship.

#### 3.2.1. Astrocytes

Protoplasmic astrocytes are the most numerous component of the striatal parenchyma. Despite their standard visualisation by detection of GFAP, most of them are GFAP-negative.

**Figure 9:**
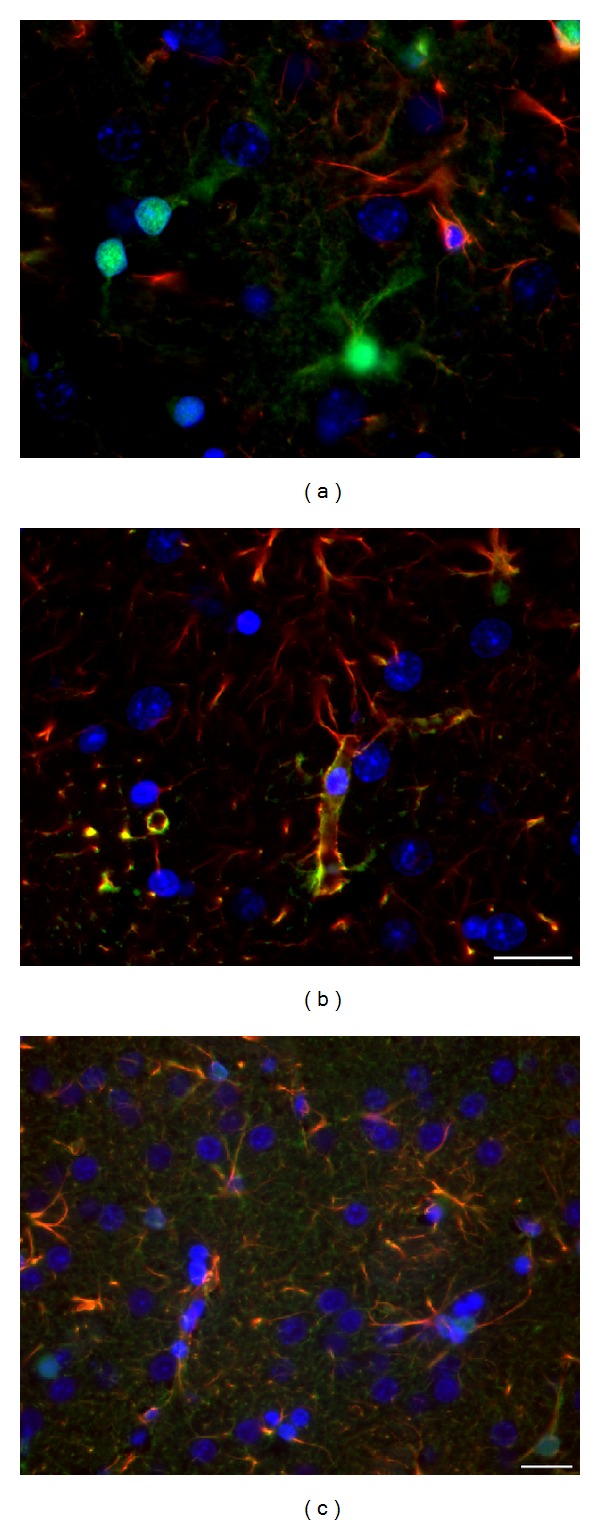
(a) In young (2-month-old) wt rats S100*β* ^+^ astrocytes are predominantly GFAP-negative; with the progression of NDP in (b) 18-month-old tgHD rats and (c) 24-month-old tgHD rats the main GFAP^+^ processes become coarser, albeit less branched, and the most distinct are their vascular end-feet (thickening of the perivascular membrane in form of “rings”); the number of not only S100*β* ^+^/GFAP^−^ but also S100*β* ^+^/GFAP^+^ astrocytes increases primarily around the vessels; the coexpression is seen mainly within the thick astrocytic processes terminating by the end-feet on the vessel wall.* Anti-GFAP (red) + anti-S100*β* (green) + DAPI (blue)*.* Bar *20 *μ*m.

**Figure 10:**
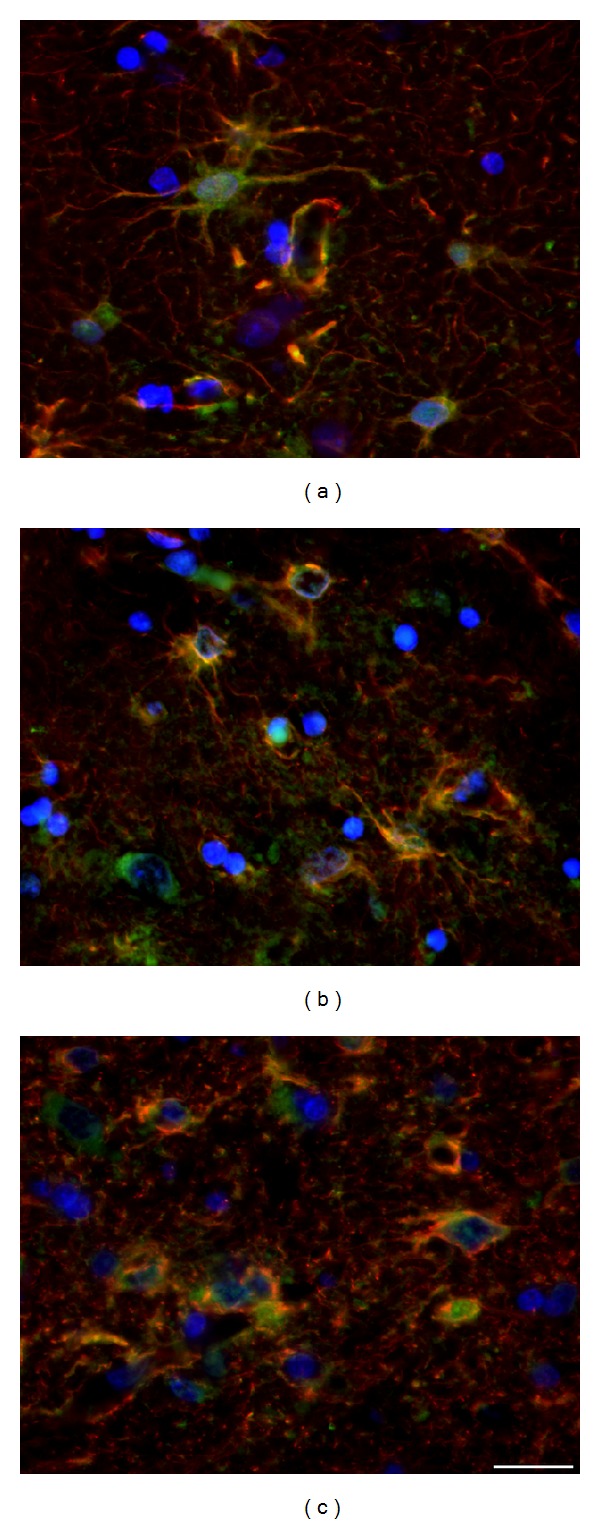
In HD human brain, significant “reactive” astrogliosis develops with the progression of NDP (b, c) in comparison with control brain (a); also the fine GFAP^+^ processes become numerous and of characteristic arborization; specific terminal swellings are in most of the densely GFAP^+^ processes; although the amount of S100*β* ^+^/GFAP^−^ is not significantly influenced by NDP, the regressive changes (shrinkage) affect also S100*β* ^+^ cells. (a) Control (♂/56), (b) HD duration 8 years, grade 3 (♂/38), and (c) HD duration 20 years, grade 4 (♀/52).* Anti-GFAP (red) + anti-S100*β* (green) + DAPI (blue)*.* Bar *20 *μ*m.

The shape of astrocytes changes during the progression of NDP; however specific prominent alterations occur only in human HD brains, where the astrogliosis gradually develops (Figures 10(b) and 10(c)). In tgHD rats, the main GFAP^+^ processes get coarser, albeit less branched. Indeed, the most distinct part of the processes (enlarged and densely GFAP^+^) becomes their vascular end-feet (Figures 9(b) and 9(c)). Hence, the main astrocytic processes and the perivascular limiting membrane are the most prominent GFAP^+^ structures in striatal parenchyma, not only in brains of tgHD rats surviving for 12−24 months (Figures 9(b) and 9(c)), but also in old wt rats, as well as in postmortem specimens of HD patients (Figures 10(b) and 10(c)). Due to only slow development of neuronal degeneration in the striatum of tgHD rats, the subsequent astrogliosis progresses also slowly—with insignificant onset after 6 months of age of tgHD rats—and becomes more distinct just in 18–24-month-old animals (Figure 9(c)). Age-related changes not only are seen in old wt rats, but also participate in progression of reactive gliosis in tgHD rats. Less (in wt rats) or more (in tgHD rats) developed striatal atrophy is manifested in senescent rats by denser accumulation of (smaller) nuclei of neurons and glia (compare Figures 2(a)–2(c)).

In HD human brains, besides the gradually increased number of reactive astrocytes, their fine GFAP^+^ processes become numerous although shorter in comparison with control brains and of characteristic arborization, forming a fine loosely arranged network, whose density increases with the progression of the disease. Moreover, we identified the specific terminal swellings in most of densely GFAP^+^ processes, whose number and also size slowly but gradually increase with the progression of HD (Figures 10(b) and 10(c)).

Despite the fact that the astrocytes, engaged in NDP, are described as “reactive,” it is necessary to point out that they are of quite different structure in comparison with typical reactive astrocytes appearing after the acute brain injury. First of all, in both HD patients and tgHD rats, generation of reactive astrocytes proceeds gradually and slowly, unlike the almost immediate appearance of reactive astrocytes after the acute brain damage. Indeed, their bodies are not significantly enlarged (hypertrophic); contrariwise, a part of them also undergoes the degeneration and they are scavenged by microglia (Figures 12(c), 13(c), and 13(d)). Additionally, enlarged GFAP^+^ vascular end-feet, which typically highlight the wall of vessels in developing NDP (Figures 9(b), 10(b), and 10(c)), and increased amount of GFAP^+^ gliofilaments forming thick bundles within the cytoplasm of reactive astrocytes belong to significant features of concomitant astrogliosis in brain of both tgHD rats and HD patients. On the contrary, we never found the reexpression of intermediate filaments nestin and vimentin, which is considerable feature of hypertrophic reactive astrocytes after the acute brain damage.

#### 3.2.2. S100*β* Protein-Positive Glia

S100*β* protein is glial-specific and it is expressed primarily by astrocytes, although not by all of them. Since this protein participates in many processes related to glial-glial and neuronal-glial crosstalk, we were interested in relevance of S100*β* expression by astrocytes in relation to developing NDP of HD phenotype. Surprisingly, the number of S100*β* ^+^ astrocytes is not markedly influenced by the progression of chronic NDP either in tgHD rats or in human HD brain. The majority of S100*β*-positive astrocytes are GFAP-negative, primarily in healthy brain. Intensely S100*β* labelled are cell bodies (incl. nuclei) and large processes terminated by end-feet. Although less prominent, expression is also in fine astrocytic processes. Since S100*β* ^+^ cells in the striatum are mainly the astrocytes, they may reflect the changes caused by the progression of NDP (Figures 9(b), 9(c), 10(b), and 10(c)), as well as by ageing process in controls. Such alterations in astrocyte immunoreactivity/morphology were primarily described on GFAP^+^ astrocytes but naturally affect all subtypes of astrocytes. In both NDP and ageing, bodies of numerous either S100*β* ^+^ or GFAP^+^ astrocytes slightly decrease in size and their processes become coarser (Figures 9 and 10). Moreover, the coexpression of S100*β* and GFAP increases, although rather differently in tgHD rats and human HD brains. In tgHD rats, the coexpression is slightly enhanced in end-feet (Figures 10(b) and 10(c)), unlike significant coexpression in both the cytoplasm of cell bodies and end-feet in human HD brains (Figures 10(b) and 10(c)). It is likely that different intensity of S100*β*/GFAP coexpression depends on a severity of the damage of striatal parenchyma which resulted, except others, in thickening of perivascular limiting membrane (i.e., vascular end-feet). Contrariwise, we could not confirm the expression of S100*β* by NG2 glia either in controls or in relation to striatal NDP.

#### 3.2.3. NG2 Glia (Polydendrocytes)

**Figure 11:**
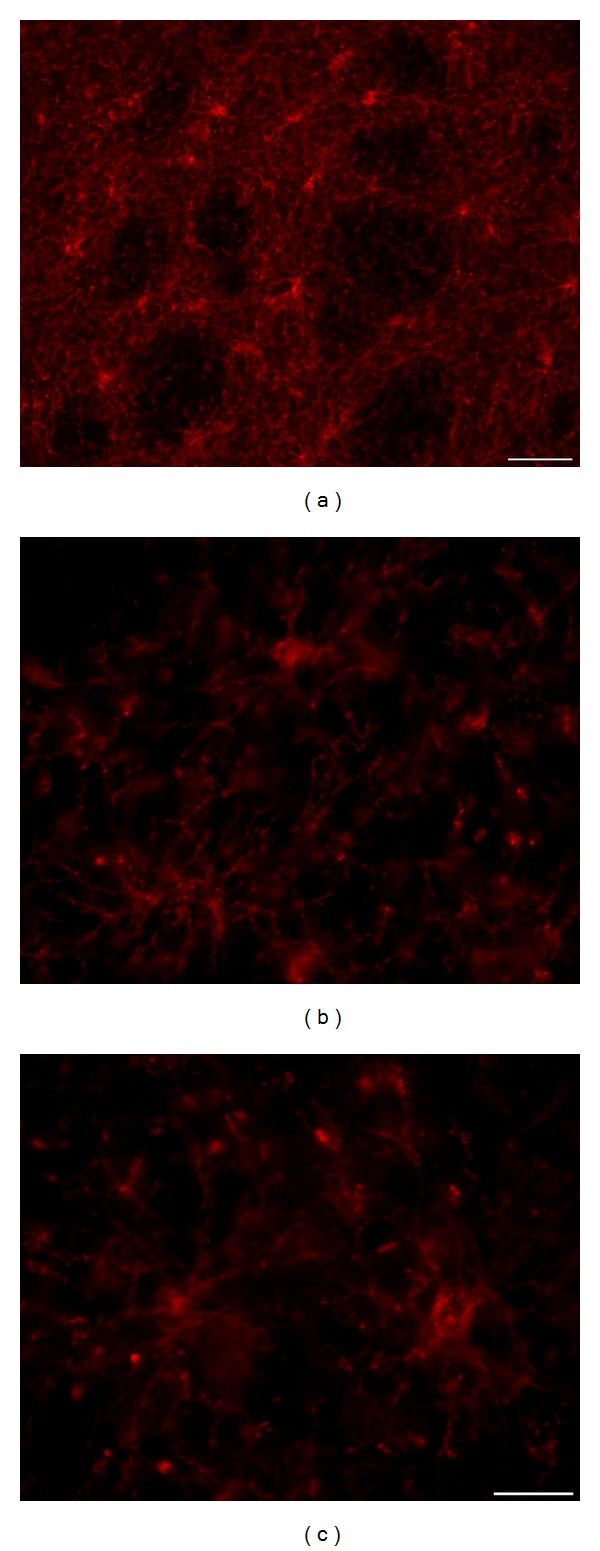
NG2 glia forms a dense 3D network, particularly prominent on thick (30 *μ*m) slices of the rat brain; there is no significant alteration in density and arrangement of this network if we compare control wt rats with age-matched tgHD rats. (a, b) 18-month-old wt rat; (c) 18-month-old tgHD rat.* Anti-NG2*.* Bar (a) *50 *μ*m* and (b, c) *20 *μ*m.

They are closely related to the development of myelinating oligodendrocytes, whose precursors are considered. We were interested in their possible alterations related to the progression of NDP in tgHD rats and also in senescence. Due to technical reasons (particularly the length of formalin fixation and the use of paraffin sections only), we were not able to detect NG2 glia cells in postmortem samples of human brains. In the rat brain, we did not prove any significant changes either in their number or in morphology in a course of the development of NDP, even in ageing process. In all examined samples, they were numerous (e.g., they outnumbered the GFAP^+^ astrocytes) and large number of their fine branched processes forms a very dense three-dimensional network throughout the entire striatal parenchyma, particularly in the grey matter (Figures 11(a)–11(c)). We also did not prove colabelling of NG2 with astrocytic markers, such as GFAP or S100*β*, which might suggest that astrocytes in response to chronic slowly developing NDP are generated in prolonged time window.

#### 3.2.4. Microglia

**Figure 12:**
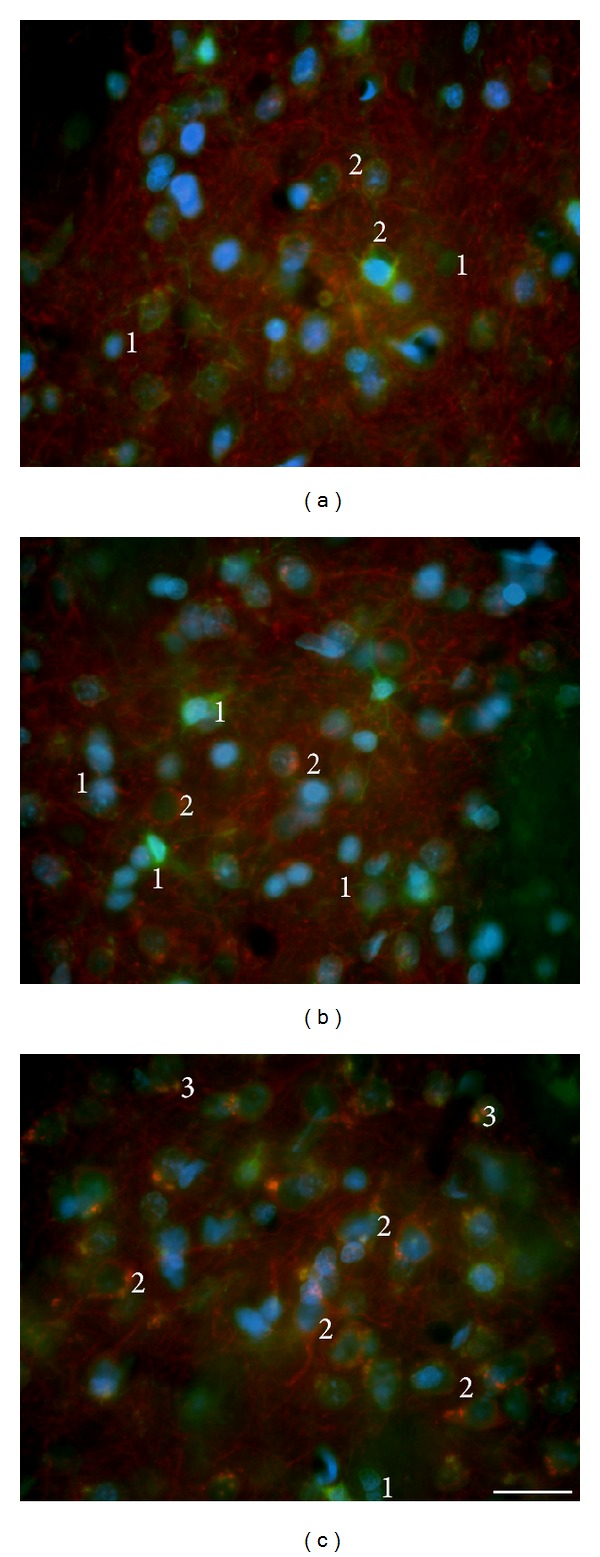
(a) Microglia* (green)* in the striatum of young (6-month-old) wt rats were mostly of small size, that is, nonactivated, although some neurons are here also scavenged (physiological degeneration); (b) the number of microglia increases quite slowly with the progression of NDP in tgHD rats; however (c) significant increase is only in the oldest animals (from 18 months of age). The double-staining with MAP2 antibody enables to document different stages of the degeneration of striatal neurons* (red)* up to the removal of their remnants; moreover, the accumulation of debris* (red)* in the cytoplasm of microglia* (green)*; (b, c) 1—inactive microglia, 2—scavenged neuron (early stage of phagocytic process), and 3—remnants of the ingested material (phagosomes) in the cytoplasm of microglia. Additionally, significant gradual reduction in the number of dendrites (i.e., rarefaction of the neuropil) with the progression of NDP is evident in aged tgHD rats. (b) tgHD—12-month-old rat; (c) tgHD—18-month-old rat.* Anti- MAP2 (red) + anti- Iba1 (green) + DAPI (blue)*.* Bar *20 *μ*m.

**Figure 13:**
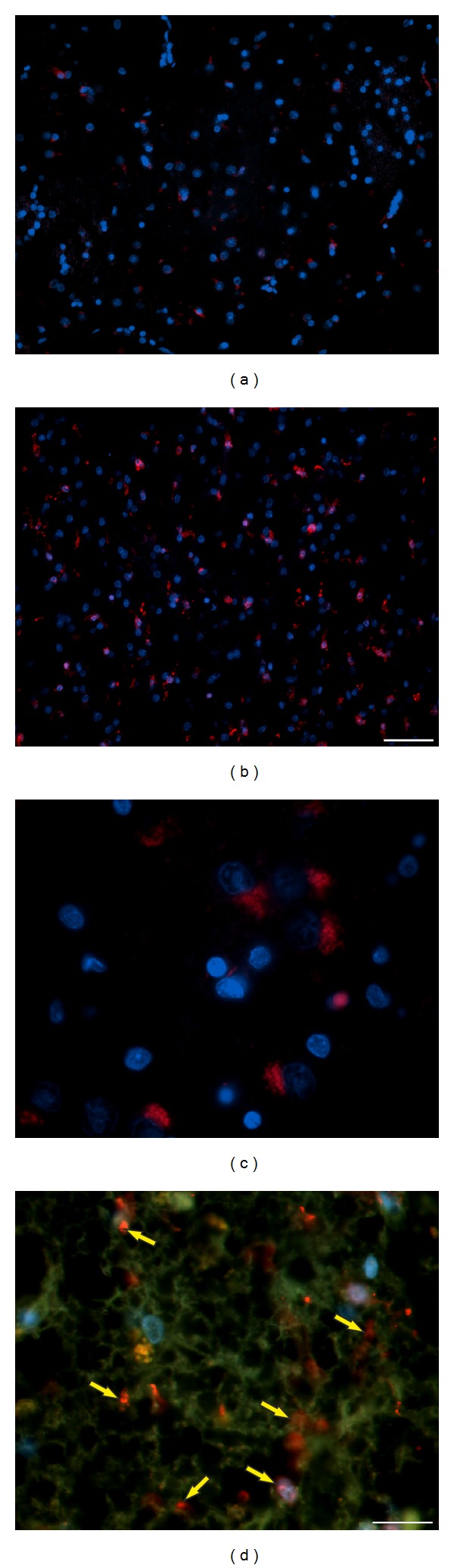
(a) In control human brain (♂/33), microglia* (red) *are mostly inactive/small (here in CN), but (b) their number and size significantly increase in advanced stage of NDP in HD brain (CN) (♀/52, HD duration 20 years, grade 4); (c) degenerated neurons within the CN are scavenged by microglia labelled with densely red stained lysosomes inside their cytoplasm (♂/38, HD duration 8 years, grade 3); (d) in advanced stage of NDP (♀/52, HD duration 20 years, grade 4), almost all neurons in CN already degenerated and they were replaced by reactive astrocytes; S100*β* ^+^ astrocytes also form a typical network; however, the neuropil is only sparse; microglia are numerous, particularly related to vessels* (yellow arrows)*.* (a, b, and c) Anti-Iba1 (red) + DAPI (blue)*;* (d) anti-Iba1 (red) + anti-S100*β* (green) + DAPI (blue)*.* Bar: (a, b) *50 *μ*m;* (c, d) *20 *μ*m.

They represent a special type of professional phagocytes occurring only in CNS, which are spread out primarily within grey matter (Figures 12(a) and 13(a)), but in lower number they are present also in white matter. In agreement with previous studies we document their upregulation with the progression of NDP, particularly in advanced stages, in both tgHD51 rats and HD brains (Figures 12 and 13), unlike their only slowly growing number in ageing control animals. In the oldest (18–24-month-old) wt control rats, their number is evidently higher, which confirms physiological increase of neuronal degeneration in aged animals. Interesting morphological findings appeared in relation of microglia (Iba1^+^) to degenerated neurons (MAP2^+^). This double-staining enables to document the consequence of stages of neuronal degeneration and removal of neurons, including the accumulation of ingested debris inside the microglial cells (Figures 12(b) and 12(c)). Indeed, glial cells (mainly astrocytes) degenerate as well and are scavenged in both rat and human brain under the pathological (e.g., Figure 13(d)) as well as physiological conditions.

In human brain, in relation to advancing NDP, the growing number of microglia is also observed (Figure 13(b)), especially in comparison with normal control brains (Figure 13(a)). However, the large number of microglia is present only in relation to degenerated and scavenged neurons; when most of striatal neurons are destroyed (i.e., in seriously damaged CN - grade 4, 20-year ongoing clinical manifestation of HD), a subsequent decrease in number of microglia is detected (Figure 13(c)). Concurrently, the majority of remaining microglia (intensely Iba1^+^) are settled close to vessels (Figure 13(d)). By contrast, in the Pu of the same sample, degenerated scavenged neurons and therefore also the microglia were still present in a large amount.

In summary, the hallmark of NDP in tgHD51 rats is a slow degeneration of striatal neurons, manifested primarily by gradual decrease in size of neuronal bodies/nuclei (with maintenance of nucleo-cytoplasmic rate) accompanied with the rarefaction of neuropil. Using the quantitative analysis, we clearly demonstrated for the first time that the turn point in the progression of neurodegenerative process in tgHD51 rats is before the end of the first year of animal age. Then, between 12 and 24 months of age, the further progression is gradual but at a slower rate, resulting in death of many neurons. Moreover, we confirmed that the development of NDP within the striatum is accompanied with gradual degeneration of cortical, particularly pyramidal neurons. We also documented significant participation of the glia, of which function in the development of NDP is irreplaceable. Most prominent is the involvement of GFAP^+^ astrocytes, particularly their transformation into the specific type of reactive astrocytes. This transformation is responsible for alterations in a structure (and therefore also in a function) of the perivascular glial limiting membrane, loosening of neuropil, and other changes. Surprisingly, we cannot confirm noticeable changes in morphology or number of NG2 glia in tgHD rats, unlike significant participation of microglia and, although less prominent, involvement of S100*β* ^+^ cells in NDP in both tgHD and human HD brains. Certainly, the entire process is potentiated by ageing changes. Our results confirm the complexity of the entire process, in which the neuron-glia crosstalk is crucial.

## 4. Discussion

The aim of our study was primarily motivated by the absence of histopathological characteristics of chronic neurodegenerative process of HD phenotype in transgenic HD51 CAG rats, which, unlike the other transgenic animal models of HD, survive up to 2 years. Moreover, this transgenic model comprises relatively smaller number (51) of CAG repeats. Both mentioned hallmarks create conditions for similarity to the late-onset form of HD. For this reason, we tried to define to which extent is possible to make a parallel between rat tgHD model and real NDP in human HD brain from histopathological point of view. On the other hand, behavioural symptoms were already widely studied on these animals* (see below)*.

Despite the fact that HD takes place exclusively within human brain and each type of existing animal models is not able to replicate completely mechanisms participating in NDP of the HD phenotype, transgenic models represent a crucial part in the field of the research on HD pathogenesis.

### 4.1. Mutant Huntingtin and Polyglutamine Deposits

It is generally known that HD is a neurodegenerative movement disorder caused by genetic mutation and morphologically characterized by progressive but selective loss of neurons, primarily within the striatum, followed by the development of reactive gliosis (e.g., [52]). Regardless of some* in vitro* studies which indicated formation of polyQ inclusions that reduce levels of mhtt and the risk of neuronal death [10, 11], it was suggested that the aberrant protein huntingtin (mhtt) with an expansion of N-terminal polyglutamine tract causes preferentially degeneration of striatal neurons in patients with HD (e.g., [6]). Moreover, a gain of a new toxic function of the mhtt results in the loss of former protective functions of wild-type htt, which ultimately leads to the death of neurons [8]. Accumulation of polyQ within the neurons and inside the neuropil is primarily described in a form of aggregates. Our findings showed fine polyQ-huntingtin-positive deposits within the nuclei of striatal neurons in control wt rats and their increase up to very dense accumulation in course of the progression of NDP in tgHD51 rats. On the other hand in cortical (particularly pyramidal) neurons, polyQ^+^ deposits were not only intranuclear but also particularly cytoplasmic. PolyQ-huntingtin antibody labels not only expanded polyglutamine (mhtt) but also wild-type (normal) huntingtin; therefore the deposits are also present in the brain of control rats. Hence, increased density of polyQ expression in tgHD51 rats is related to increased number of glutamine repeats, that is, to the accumulation of mhtt. Htt is particularly not only spread within the cytoplasm of neuronal bodies and dendrites [7] but also present in the nucleus [53]. Mhtt is present in both nuclear and cytoplasmic compartments, extended polyQ aggregated in the cytoplasm and then it is transported to the nucleus [54]. Increasing concentration of the mhtt in the nucleus accelerates the onset and progression of NDP; however, also extranuclear polyQ might contribute to the initiation of NDP [55]. The same but almost exclusively intranuclear polyQ deposits were also found in glial cells in both brain regions. Accumulated mhtt causes the dysfunction of astrocytes, particularly in relation to glutamate uptake, since it decreases the expression of glutamate transporters. This impairment may promote vulnerability, especially of striatal medium-sized spiny neurons, to the excitotoxic damage and their degeneration (e.g., [12, 13]). Astrocytes expressing mhtt transform into reactive phenotype, which is characterized by markedly decreased expression of glutamate transporters (GLAST and GLT-1) and glutamate uptake. These alterations appear already in early stages of HD (grade 0) and progress in a course of the disease. Therefore, it was suggested that the presence of mhtt in astrocytes and their conversion into reactive glia may contribute to HD pathogenesis [13].

### 4.2. Reactive Astrogliosis

Reactive astrogliosis is a gradual continuous process of progressive alterations in gene expression and cellular changes. The intensity and extent of the reactive gliosis are determined primarily by signals from damaged cells (e.g., [56, 57]). Up to now, the origin and the way by which the increased number of astrocytes appears in damaged CNS are not fully elucidated and still remain a matter of intensive debate. Considering various types of CNS disorders, marked differences in development of astrogliosis should be taken into the account. During acute CNS injury, reactive gliosis develops within two days and reaches its maximum 1-2 weeks following the insult, while chronic injuries, such as neurodegenerative diseases, are characterised by slow development of astrogliosis, which may last even several years in humans [58].

Owing to the fact that only very low (if any) response of neural progenitors to the CNS damage is found in all nonneurogenic regions, the source for generation of GFAP^+^ reactive astrocytes remains under discussion—two plausible possibilities are proposed: (1) transformation or dedifferentiation from resident astrocytes which has been confirmed after different neurotoxic lesions in rodent brain (e.g., [59, 60]) or the activation of “quiescent” protoplasmic astrocytes (a subset of GFAP^+^ astrocytes) acquiring stem cell properties in adult mouse cerebral cortex after acute injury [61]; (2) differentiation of glial progenitors expressing Olig2 and NG2 [62, 63]. For that reason we were surprised with the absence of any obvious involvement of NG2 glia and principally also S100*β*-positive astrocytes in reactive gliosis. On the contrary, both mentioned possibilities were described in relation to the acute lesion of rodent brain. How the generation of new reactive astrocytes occurs in human brain suffering from chronic neurodegenerative process or in brain of transgenic animals modelling neurodegenerative diseases, as well as the participation of NG2 in the progression of NDP, particularly in HD patients, remains unclear.

It is evident that the development of reactive gliosis and the alterations in astrocyte morphology are conditioned by the intensity and rate of neuronal degeneration in the context of the neuron-glia relationship [64]. Our findings also document significant difference between typical reactive astrocytes, which change their phenotype in reaction to the acute brain injury or excitotoxic lesion, used formerly as an animal model of HD (e.g., [58, 65]) and “reactive” astrocytes developing during slow progression of chronic neurodegenerative process of HD phenotype. The most conspicuous are changes in expression of GFAP, that is, in the number and arrangement of gliofilaments (the principal astrocytic intermediate filaments) within the cytoplasm. Reactive astrocytes become more stellate with only few but coarse main processes, especially those terminating as the end-feet on the vessel wall. They are intensely labelled for GFAP, due to not only increased amount of GFAP^+^ gliofilaments but particularly their accumulation to thick bundles (e.g., [34, 64]). This alteration is evident in both tgHD and human HD brains and progresses with the development of NDP. Furthermore, this process occurs not only in HD, but also in other neurodegenerative diseases, such as Parkinson's (e.g., [48, 56]) and Alzheimer's disease (e.g., [66]). Indeed, similar changes, although to a lesser extent, are typical for astrocytes in a course of ageing in both human and rodents (e.g., [66]). Significant for those “reactive” astrocytes involved in NDP is also the absence of reexpression of other intermediate filaments, nestin and vimentin (abundantly expressed in immature astrocytes), whose adaptation is characteristic for immediate activation of astrocytes not only in case of the acute brain injury (e.g., the formation of the glial scar) but also in neurotoxic models of HD [64]. Moreover, these astrocytes do not become markedly hypertrophic (e.g., [34]).

### 4.3. Neuronal-Glial-Vascular Unit

Except for others, very important is also the relationship of astrocytes to the vessels. Our previous findings documented the thickening of end-feet (and this way of the perivascular limiting membrane), which markedly outlines vessel walls forming typical “rings” with the progression of NDP in excitotoxic rat model of HD [64]. Our findings also demonstrate that those coarser end-feet not only are intensely GFAP-positive but also frequently coexpress S100*β* protein in both tgHD rat and HD human brains. These gradual alterations in the architecture of the vascular end-feet might contribute to the restriction of transport or diffusion across the blood-brain interface (e.g., [31, 56]). Additionally, the specific enlargement at the tips of astrocytic processes in a form of intensely GFAP-positive “terminal swellings” was found in advancing NDP in human HD brains (grades 3 and 4). However, there is no evidence, which structure in neuropil is in contact with these modified endings. Hypothetically, they might belong to those extensions that lost contact with any opposite structure. In agreement with the other authors, we have indicated participation of ageing changes [67] in the above-mentioned alterations of astrocytic morphology in the oldest groups of control animals.

### 4.4. CNS Phagocytes: Microglia and Astrocytes

It is also well known that astrocytes are involved in degradation of different metabolites and toxic products under the normal conditions. Their ability to function as phagocytes is then enhanced under the pathological conditions (e.g., [68]). It was proposed that chronically activated astrocytes and microglia can damage neurons via release of highly toxic products (e.g., [66]) and also that microglia play an important role in pathogenesis of Parkinson's disease. This latter suggestion is based on the fact that, with progressive neuronal loss, increasing amounts of *α*-synuclein and neuromelanin accumulate in the extracellular space due to dysfunction in microglia phagocytic ability [49]. In general, debris accumulation is supposed to be one of key features of the progression (if not direct initiation) of neurodegenerative diseases.

### 4.5. NG2 Glia

To our surprise, we did not find any significant alterations in number and morphology of NG2 polydendrocytes in the presence of pathology. They formed a typical dense network in all tested groups of tgHD and wt rats. Due to technical reasons, we were unfortunately not able to follow them in postmortem specimens of human brains, either with HD or intact. It is probably the main reason why the involvement of NG2 glia in NDP has not been reported yet in brains of HD patients. The elegant study employing postmortem specimens of human brain tissue with multiple sclerosis (MS) lesions or glioma (using both frozen and paraffin sections) was published by Staugaitis and Trapp [44]. They also discussed difficulties with detection of NG2 glia in routinely processed surgical and autopsy tissue. However, no obvious decrease in density of NG2 glia was found in the grey matter of cerebral cortex with MS lesions [44, 69]. Indeed, most of other studies which reported the relationship of NG2 glia to human CNS pathology are based only on* in vitro* experiments (e.g., [70]).

### 4.6. Participation of Neurons and Glial Cells in Neurodegenerative Process

It is obvious that glia, particularly GFAP-positive astrocytes, play a critical role in the progression of NDP. Nevertheless, their involvement is always in a context of changes affecting simultaneously all components—cellular and noncellular—of the nervous tissue microenvironment. Also microglia is suggested to be an important player in complex response of nervous tissue to the chronic damage, principally by the activation of cascade of the immune response.

The evaluation of the contribution of GFAP^+^ astrocytes to neurodegenerative process remains still unclear, albeit their dual role—neuroprotective and neurodegenerative—was already mentioned by some authors (e.g., [27, 56, 71]). On the one hand, astrocytic degeneration accompanying degeneration of the neurons obviously participates in the processes resulting in disintegration of the nervous tissue (loosening of the neuropil, thickening of perivascular glial limiting membrane, and others). On the other hand, there are some indications that changes in astrocyte character and function belong to neuroprotective mechanisms in damaged nervous tissue.

Regardless of the interesting and challenging findings related to the different types of glial cells, the degeneration and loss of neurons, primarily within the striatum, accompanied with loosening of the neuropil still remain the hallmark of HD. However, the disintegration of the neuropil not only results from gradual reduction of neuronal processes, but also includes the alterations of the glial cells. Indeed, regression of the neuropil represents the progressive disruption of the striatal microenvironment, including the vascular niche, essential for the viability and functioning of all the components of striatal tissue, which even more contributes to worsening of the entire process. The reduction of the grey matter results in a severe brain (primarily striatal) atrophy in advanced NDP.

### 4.7. Rats Transgenic for HD

Transgenic HD51 CAG rats were used also by other research groups, mainly to validate changes in their behaviour, which indicates impaired striatal function (e.g., [9, 21–24]). The tests proved an early learning and memory impairment (starting between 9 and 12 months) and delayed motor deficits (with the onset around 12 months); moreover, these deficits develop prior to any diagnosed striatal dysfunction. Additionally, age- and genotype-dependent changes in psychomotor performance and in the frequency of choreiform movements were proved in homozygous, less heterozygous tgHD rats in comparison with controls at the age of 15 months and particularly at 20 months [22].

Concerning morphological evaluation, it was shown that polyQ aggregates appear within the striatum of tgHD rats at 12 months of age [9, 23] and the density of their accumulation increases with age, which is consistent with our observations. Furthermore, the quantitative analysis of total striatal volume was carried out in animals from 3 to 15 months of age (using stereological method), which confirmed significant gradual decrease in striatal volume from 12 to 15 months in tgHD rats compared with wt littermates [9] or in 12-month-old rats (compared with 6-month-old and wt rats), respectively [24]. The only reference of Kántor and coauthors [24] provides quantitative analysis of total numbers of neurons and neuronal density in the striatum and a part of frontal cortical layer V in groups of 6- and 12-month-old tgHD51 and wt rats. The results related to the striatum are comparable with our observations. However, the hallmark of neurodegenerative process in the brain of tgHD rats is gradual decrease in size of striatal neurons, unlike the significant decrease in their number in postmortem samples of HD brains. Therefore, we used median diameter of neuronal nuclei to document the onset and progression of NDP in 5 groups of tgHD rats and age-matched wt rats. Nevertheless, to analyse the density of striatal neurons (i.e., their number per defined square unit) becomes rather problematic in the oldest rats (1.5–2 years old) due to significant shrinkage of the striatum. Kántor and coauthors also concluded that, unlike striatal neurons, the cortical neurons (in layer V) did not reveal significant degeneration in 12-month-old tgHD rats. We documented the presence of significant number of hyperchromic degenerated neurons in this age in comparison with age-matched control rats, which is consistent with changes within the cortex of human HD brain [2]. Moreover, already in 6 months old tgHD rats, degenerated cortical neurons appeared. On the other hand, in both tgHD51 rats and HD patients, the extent of neuronal degeneration in the cortex never reaches extensive degeneration within the striatum; therefore, it cannot be accompanied with reactive gliosis.

## 5. Conclusions

In this study, histopathological features, significant for alterations in human brains suffered from HD and brains of tgHD51 CAG rats, were compared. The significant difference is primarily in the intensity of neuronal degeneration which, even in the oldest rats, does not reach the severity of the damage characteristic for advanced stages of HD in humans. In tgHD51 rats, quite large amounts of striatal neurons display only gradual decrease in their size and do not fully degenerate and disappear until the death of animal (at about 2 years of age). Therefore, also concomitant reactive astrogliosis is not extensive in old tgHD rats, unlike the advanced stages of HD in humans. On the other hand, striatal atrophy (the reduction of grey matter) develops gradually, and it is distinguishable from 12 months of age in tgHD rats, manifested primarily by compensatory enlargement of lateral brain ventricles. The reason for striatal shrinkage is the same in rats and humans; it originates from the gradual degeneration of neurons resulting in rarefaction of the neuropil including the alterations in the density and character of synapses. Our data also support the view that all types of CNS glial cells play an important, irreplaceable role in neurodegenerative process. There are different indications that changes in their character and function try to balance the development of serious irreversible damage of the striatum as long as possible. However, when this battle exceeds all their capacities, the system collapses and their involvement becomes detrimental, finally resulting in death of an individual. All the available results in this field directly or indirectly confirm that the response of nervous tissue to any damage is always complex (moreover in relation to the entire organism) and therefore has to be considered in this context.

To the best of our knowledge, our findings are the first to document that tgHD51 CAG rats can be used as a valid animal model for detailed histopathological studies related to HD in human. On the other hand, since HD occurs only in humans and we are not able to reproduce fully this process in animals till now, the interpretation of all findings has to be always carefully weighted and sufficiently critical.

## Figures and Tables

**Table 1:** Antibodies used.

Antibody	Host	Dilution	Source	Report
Nestin	Rat monoclonal anti-mouse	1 : 4	DSHB	Progenitor cells marker
Nestin	Mouse monoclonal anti-human	1 : 200	Millipore	Progenitor cells marker
*β*-III tubulin	Mouse monoclonal	1 : 20	Exbio	Neuronal marker
MAP2	Mouse monoclonal	1 : 500	Sigma-Aldrich	Neuronal marker
MAP2	Rabbit polyclonal	1 : 700	Millipore	Neuronal marker
NeuN	Mouse monoclonal	1 : 100	Millipore	Marker of mature neurons
Synaptophysin	Mouse monoclonal	1 : 20	Dako	Marker of neuronal synapses
Vimentin (Cy3 conjugated)	Mouse monoclonal	1 : 100	Sigma-Aldrich	Astrocyte and radial glia marker
NG2	Rabbit polyclonal	1 : 400	Millipore	Oligodendrocyte precursor and pericyte marker
APC	Mouse monoclonal	1 : 200	Calbiochem	Oligodendrocyte and astrocyte marker
GFAP	Mouse monoclonal	1 : 400	Sigma-Aldrich	Astrocyte marker
GFAP	Rabbit polyclonal	1 : 400	Dako	Astrocyte marker
S100*β*	Mouse monoclonal	1 : 1000	Sigma-Aldrich	Astrocyte marker
S100*β*	Rabbit polyclonal	1 : 300	Dako	Astrocyte marker
Iba1	Mouse monoclonal	1 : 300	Millipore	Microglia and macrophage marker
PolyQ-huntingtin	Mouse monoclonal	1 : 20000	Millipore	Polyglutamine inclusions marker

**Table 2:** Results of Kruskal-Wallis Multiple-Comparison *Z*-Value Test (Dunn's Test) of the groups of rats: “young_wt” and “young_tgHD” are groups of rats 3 and 6 months (0–6 months) old; “old_wt” and “old_tgHD” are groups of rats 12, 18, and 24 months old (>6 months). Asterisk indicates significantly different pairs of groups (Bonferroni test: medians are significantly different if *Z*-value is > 2.6383). Statistically significant is a difference in the median diameter of neuronal nuclei in “old_tgHD” group in comparison with all other evaluated groups.

Group	young_tgHD	young_wt	old_tgHD	old_wt
young_tgHD				
young_wt	0.2844			
old_tgHD	4.9557∗	4.5959∗		
old_wt	1.813	1.4646	4.5135∗	
